# Double naso-enteric tubes stenting in afferent limb syndrome with concomitant proximal efferent limb obstruction after loop gastrojejunostomy bypass: a case report

**DOI:** 10.1093/jscr/rjae768

**Published:** 2024-12-06

**Authors:** Jirat Leelapatanadit, Rawat Waratchanont, Wichitra Asanprakit, Viriya Kaewkangsadan, Sukchai Satthaporn

**Affiliations:** Department of Surgery, Phramongkutklao Hospital, Thung Phaya Thai, Ratchathewi, Bangkok 10400, Thailand; Department of Surgery, Phramongkutklao Hospital, Thung Phaya Thai, Ratchathewi, Bangkok 10400, Thailand; Department of Surgery, Phramongkutklao Hospital, Thung Phaya Thai, Ratchathewi, Bangkok 10400, Thailand; Department of Surgery, Phramongkutklao Hospital, Thung Phaya Thai, Ratchathewi, Bangkok 10400, Thailand; Department of Surgery, Phramongkutklao Hospital, Thung Phaya Thai, Ratchathewi, Bangkok 10400, Thailand

**Keywords:** naso-enteric tube, afferent limb syndrome, efferent limb obstruction, loop gastrojejunostomy complication

## Abstract

Endoscopic or fluoroscopic guided naso-enteric placement for stenting and decompression has been used in mechanical enteric limb obstruction after gastrectomy or gastric bypass surgery. However, the use of double naso-enteric tube for treatment of multiple enteric limbs obstruction has not been described to date. We present a 61-year-old female with afferent limb syndrome with concomitant efferent limb obstruction which caused by kinking of anastomosis after loop gastrojejunostomy for benign gastric outlet obstruction. Two naso-enteric tubes were placed in efferent limb and afferent limb by endoscopic and fluoroscopic guidance. The patient was able to resume oral intake after 2 weeks of tube placement.

## Introduction

Afferent and efferent limb syndromes are common complications in patients whom undergo gastric resection or gastro-enterostomy bypass. The etiologies include kinking, adhesions, internal herniation, volvulus, stenosis, inflammation, enterolith and recurrent cancer [1, 2]. In a very rare condition, afferent loop syndrome can be concomitantly occurred with proximal efferent limb obstruction [2, 3]. There are various treatment options including surgery, endoscopic treatment and conservative treatment. We present a case of afferent limb syndrome with concomitant efferent limb obstruction which caused by kinking of anastomosis after loop gastrojejunostomy for benign gastric outlet obstruction, treated with double naso-enteric tubes insertion for dekinking and stenting.

## Case report

A 61-year-old female with history of truncal vagotomy with loop gastrojejunostomy for gastric outlet obstruction due to chronic duodenal ulcer 1 month ago presented with epigastric abdominal pain and vomiting after meal for 5 days. On examination, she had marked epigastric abdominal distension. Abdominal radiography showed severe distended stomach and gasless abdomen.

**Figure 1:**
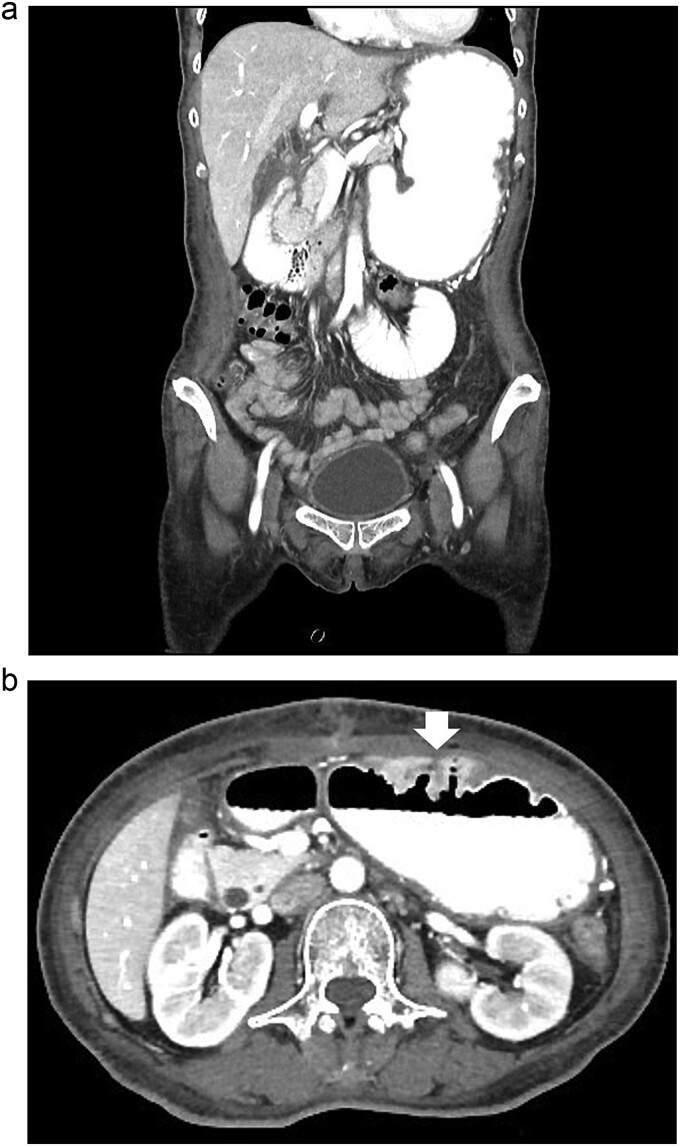
The CT scan of abdomen: (a) coronal view showed dilated stomach and afferent limb, (b) axial view showed gastrojejunostomy anastomosis angulation and kinking (arrow).

**Figure 2:**
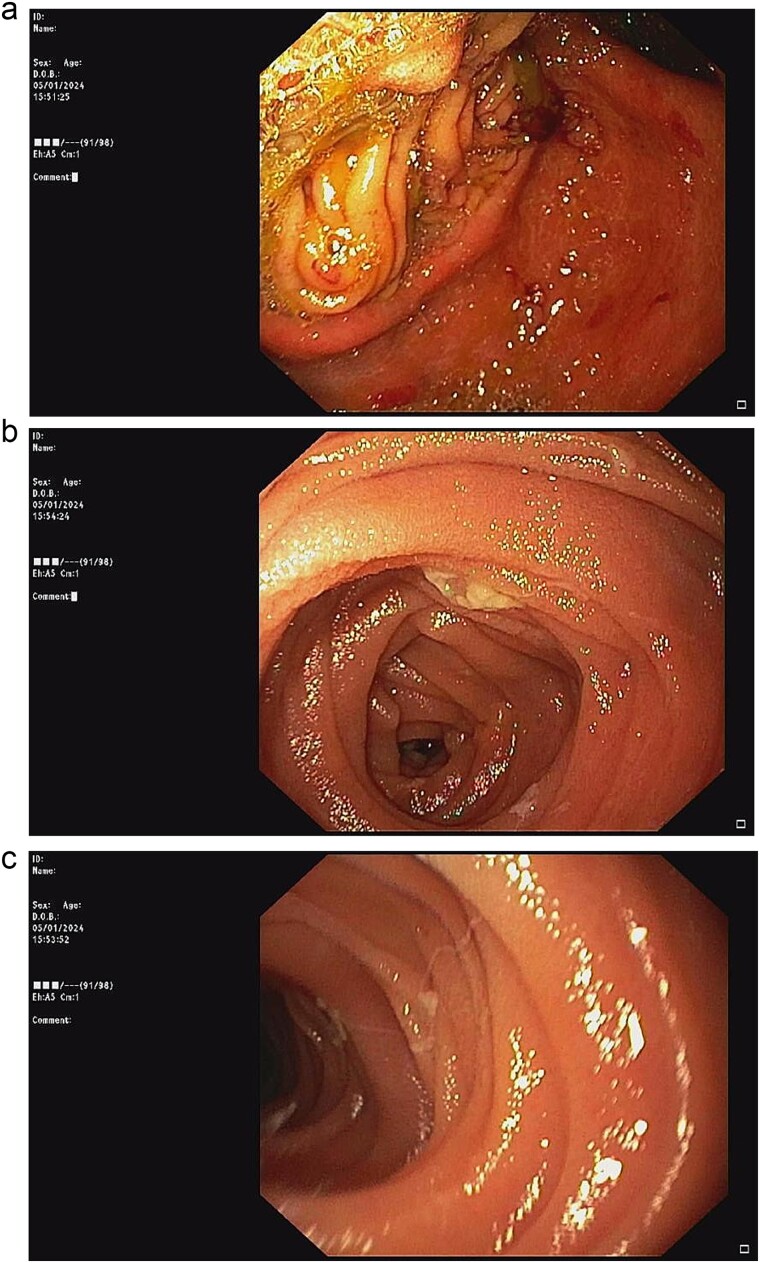
Upper endoscopic findings: (a) patent gastrojejunostomy anastomosis, (b) afferent limb distal to the angulation, (c) efferent limb distal to the angulation.

According to these clinical and radiological finding, concern of recurrent gastric outlet obstruction has been raised. Therefore, the computed tomography (CT) scan of the abdomen was performed and showed gastric and afferent limb dilatation, no oral contrast filling in efferent limb and collapsed remaining small bowel loop just distal to gastrojejunostomy anastomosis (Fig. 1). The CT scan findings was afferent limb syndrome associated with efferent limb obstruction due to gastrojejunostomy anastomosis angulation and kinking. The patient was rehydrated with intravenous fluid. Nasogastric tube was placed for decompression. After 5 days of conservative treatment, the abdominal distention was improved but the content from nasogastric tube was continuingly 800–1000 ml per day. Upper endoscopy under general anesthesia was performed for definite diagnosis and chance for endoscopic treatment. It showed patent gastrojejunostomy anastomosis but the both proximal afferent and efferent limb were angulated. Endoscopic dekinking was performed by gentle passage of the endoscope tip across the angulated segment until the intraluminal intestinal segment distal to the obstruction point was reached (Fig. 2).

**Figure 3:**
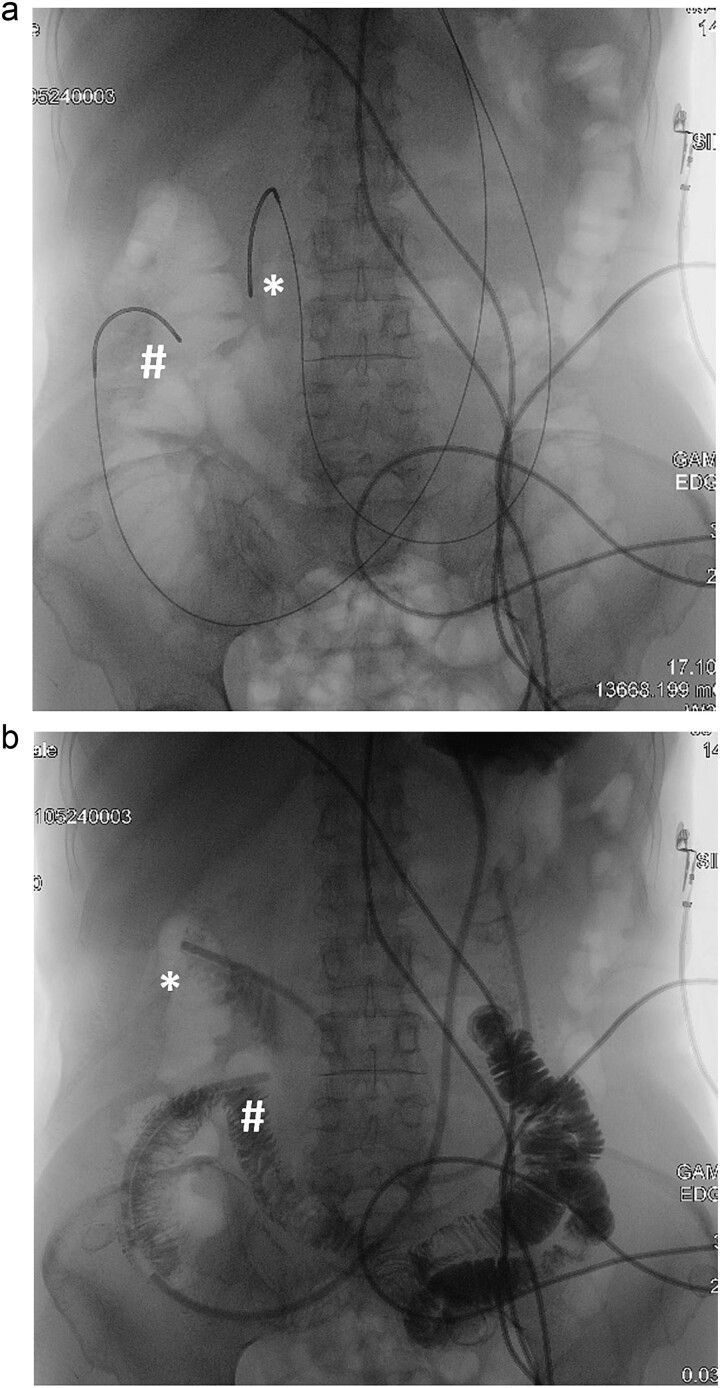
Fluoroscopy and contrast study: (a) Savary-Gilliard guidewires were placed, (b) naso-enteric tubes were placed (*: afferent limb, #: efferent limb).

Double naso-enteric tubes was placed for stenting, enteric feeding and decompression (one was in the distal efferent limb, the other was in the proximal afferent limb). Savary-Gilliard guidewires were placed through-the-scope, across the angulated point. The endoscope was then totally withdrawn, leaving the guidewire in place. Naso-enteric tubes were threaded over the guidewire across the obstructed segment by using two 16-French single lumen plastic nasogastric tube (125 cm in length). The position of the tubes was confirmed by fluoroscopy and contrast study (Fig. 3).

**Figure 4:**
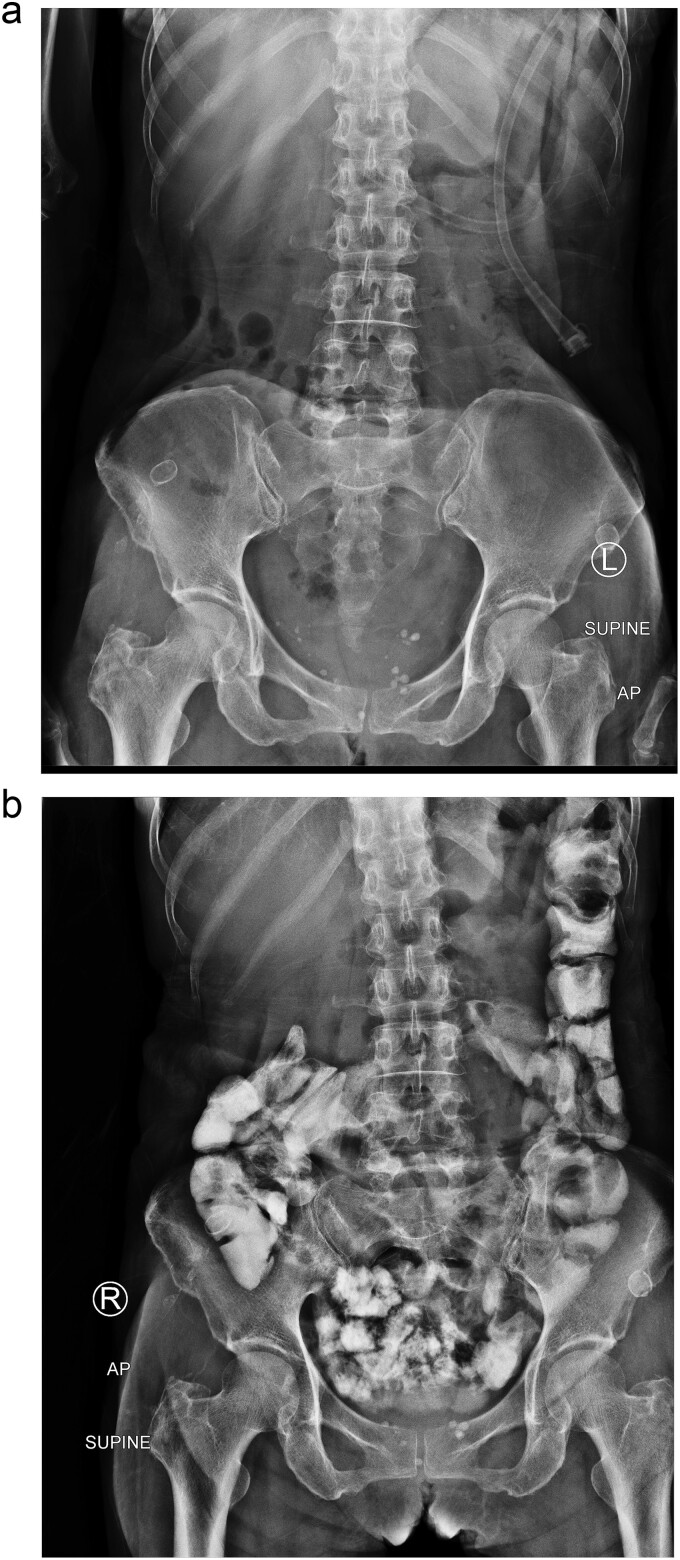
Two weeks of conservative treatment: (a) abdominal plain film, (b) oral contrast study.

After 2 weeks of conservative treatment, the content from afferent limb decreased to 100–200 ml per day. Subsequently abdominal radiography showed collapsed stomach and normal bowel gas pattern. Oral contrast study revealed that contrast passed through small bowel and large bowel in 6 hours (Fig. 4). The patient was able to resume an oral diet and discharge at hospital admission Day 25th. On 3-mo follow-up, the patient could have solid food without any symptom.

## Discussion

Afferent limb syndrome is a mechanical obstruction of afferent limb or biliopancreatic limb whereas efferent limb syndrome is a mechanical obstruction of efferent limb or enteric limb. These two conditions can occur after gastric resection or gastro-enterostomy bypass, while afferent limb syndrome is more common [4]. Infrequently, these 2 conditions can happen simultaneously when obstruction site is at or near gastrojejunostomy anastomosis [5]. The usual causes of these syndromes during the early postoperative period are anastomotic edema and kinking [3]. In this case, we hypothesized that the cause of anastomotic kinking was from delay gastroparesis after previous gastric outlet obstruction together with improper previous operative procedure.

There are various treatment options including surgery, endoscopic treatment and conservative treatment. Complete obstruction, strangulation, perforation and failure of conservative treatment require surgical treatment. Recently, a few successful endoscopic treatments have been reported including endoscopic decompression and metal [5] and double-pigtail stent placement [3]. Endoscopic small-bore naso-jejunal tube stenting across the occlusion segment in patients with partial jejunal limb obstruction during the early postoperative period of gastric related surgery has been reported with acceptable outcomes [2]. For this patient, two naso-enteric tubes were required. One in efferent limb was for enteric feeding whereas the other one in afferent limb was for decompression of stomach together with afferent limb. Both tubes were for stenting the proper angulation of gastrojejunostomy anastomosis.

Disadvantages of this method are discomfort from tubes irritation of both nostrils and long duration of treatment. In the future, further research is necessary to determine efficacy, quality of life and optimal duration of this treatment.
